# Mechanical Properties of Polymer-Based Blanks for Machined Dental Restorations

**DOI:** 10.3390/ma14237293

**Published:** 2021-11-29

**Authors:** Lucian Toma Ciocan, Jana Ghitman, Vlad Gabriel Vasilescu, Horia Iovu

**Affiliations:** 1 Department of Prosthetics Technology and Dental Materials, “Carol Davila” University of Medicine and Pharmacy, 050474 Bucharest, Romania; tciocan@yahoo.com (L.T.C.); vgvasilescu@yahoo.com (V.G.V.); 2 Advanced Polymer Materials Group, University Politehnica of Bucharest, 011061 Bucharest, Romania; horia.iovu@upb.ro; 3 Academy of Romanian Scientists, 050094 Bucharest, Romania

**Keywords:** restorative dental materials, CAD/CAM technology, dental tissue, micro-mechanical properties

## Abstract

The tremendous technological and dental material progress led to a progressive advancement of treatment technologies and materials in restorative dentistry and prosthodontics. In this approach, CAD/CAM restorations have proven to be valuable restorative dental materials in both provisional and definitive restoration, owing to multifarious design, improved and highly tunable mechanical, physical and morphological properties. Thus far, the dentistry market offers a wide range of CAD/CAM restorative dental materials with highly sophisticated design and proper characteristics for a particular clinical problem or multiple dentistry purposes. The main goal of this research study was to comparatively investigate the micro-mechanical properties of various CAD/CAM restorations, which are presented on the market and used in clinical dentistry. Among the investigated dental specimens, hybrid ceramic-based CAD/CAM presented the highest micro-mechanical properties, followed by CAD/CAM PMMA-graphene, while the lowest micro-mechanical features were registered for CAD/CAM multilayered PMMA.

## 1. Introduction

The main goal of restorative dentistry consists of the design and manufacturing of advanced dental materials with improved structural, biological and aesthetical features that can maintain the tissue integrity and replace the damaged dental tissue, ideally mimicking the natural appearance and performance of the replaced tissues, when used as restorative materials in a particular dental tissue clinical problem [1,2]. Considering the complex structure and functions of dental tissue and its great contribution to the quality of life (in eating, speaking, swallowing and proper breathing), the features and performances of restorative materials need to satisfy various demands starting from high flexibility essential for impression materials to high stiffness important in crowns and fixed dental prostheses [1].

Over time, different types of materials, e.g., metals, polymers, ceramics and composites [3], were manufactured through various methods, e.g., polymerization, casting and porcelain densification by sintering [4] to design new restorative materials with improved mechanical properties and proper characteristics for both provisional and definitive restoration.

It is documented that the performance of a dental material in the rehabilitation of aesthetic and functional restorations is driven by the best compromise between the local anatomical deformations and the design versatility and ability of the implanted material to satisfy functional and aesthetic demands without compromising its longevity [5]. In some clinical scenarios, where the implant placement can be limited by anatomical conditions (e.g., either bone deficiencies, the presence of anatomical structures or both, posterior edentulous area) and complex dental treatments are required (e.g., surgical procedures) which may lead to different complications and morbidity as well as increasing costs, the use of fixed dental prostheses with cantilever extensions that allow a more straightforward rehabilitation of these edentulous areas, still remains a long-term reliable treatment option with potent clinical, radiographic and aesthetic outcomes [6]. In a complex study, Schmid and co-workers [7] demonstrated the reliability of long-term treatment of implant-supported fixed dental prostheses with cantilever extension (FDPCs) by investigating the clinical and radiographic outcomes of implant-supported FDPCs with a cantilever extension of one premolar unit in posterior areas after a function time of more than 10 years. A 96.2% survival rate was noted of implants supporting FDPCs in posterior areas of maxilla and mandible with minimal marginal bone level changes, while the loss of retention, which represented the most frequent complication, was observed in 34.6% of patients. Further, in another study, the same research team evaluated the clinical and radiographic performances of single-unit crowns with cantilever extensions (SCCs) implanted in the same posterior areas after a loading time of at least 10 years [8]. In this case the maximum (100%) survival rate of implants supporting SCCs along with the minimal marginal bone level changes were registered, proving the long-term soundness of these restorative dental materials. Moreover, in a comparative retrospective study, Roccuzzo et al. [9] demonstrated the validity and potency of using single-implant-supported 2-unit cantilever FDP instead of two adjacent implants in anterior sites when the available mesiodistal space is limited.

**Figure 1:**
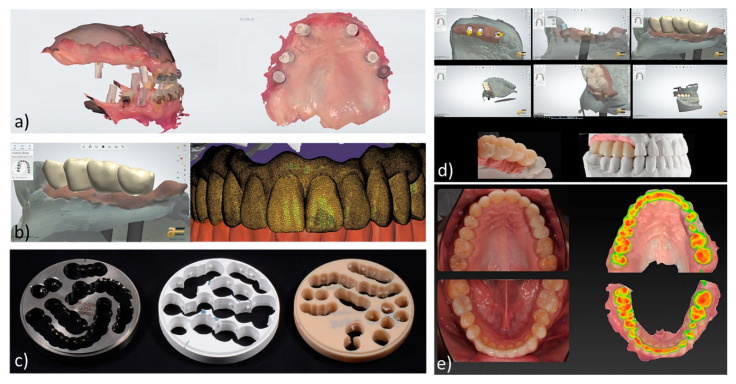
Clinical steps in CAD/CAM provisional restorations and their clinical wear analysis: (**a**) oral scanning of 3D implants position and surrounding tissues; (**b**) CAD—design of dental prosthesis; (**c**) CAM blanks: alloys (left), presintered zirconia (center), polymer (right); (**d**) CAD—design and machined final product of stone-cast oral replicas; (**e**) wear analysis after clinical use.

The emergence of computer-aided design/computer-aided manufacture (CAD/CAM) technology in dentistry has significantly improved the production of different restorative dental materials [10,11], as alternative aesthetic restorations to conventional laboratory processed materials [12]. Generally, CAD/CAM systems consist of a computer connected to a software capable of collecting and processing the information and then transferring it to a production technology, usually a milling process, that transform the data into the final product [13]. The designing concept of CAD/CAM restorative dental materials in clinical practice is presented in Figure 1.

The benefits associated with the CAD/CAM approach, such as cost-efficient personalized materials, high quality and reproducibility, almost defect-free structure, improvement in precision and planning, high efficiency, possibility to store the data of a standardized chain of production, industrially prefabricated and controlled materials and increased popularity in comparison with conventional techniques [13,14]. Moreover, many manufacturing restorative dental companies use the CAD/CAM approach, either in the dental practice, dental laboratory or in manufacturing centers.

Besides the mechanical and morphological features, accurateness of restorative materials represents another critical parameter, with important functions in the protection of connective tissue, bacterial contamination and preservation of periodontal tissues [15]. Supplementary, imperative particularities, such as prevention of the rotation of the tooth from its normal position and maintaining aesthetics and oral functions (mastication and speech), are also linked to the precision of restorative material. It has been reported that CAD/CAM can solve these challenges; the CAD/CAM composite restorative material showed better marginal accuracy than directly fabricated composite [16].

Moreover, the literature reports that CAD/CAM composite restorations present low abrasiveness being more flexible and are less fragile compared to glass ceramics. Besides, these materials can be easily modified or repaired having appropriate stress absorbing features. The efficiency of CAD/CAM restorative materials can be evaluated by laboratory-testing various mechanical, bonding or morphological parameters.

The purpose of this research study is to comparatively analyze different polymer CAD/CAM materials from a micro-mechanical properties point of view and to elect the most suitable material for its application. The micro-mechanical properties of all materials were investigated by nanoindentation method. From the load-displacement curve recorded for each material, important micro-mechanical parameters, e.g., micro-elastic modulus, micro-hardness and continuous stiffness were then extracted and comparatively analyzed.

## 2. Materials and Methods

The commercial CAD/CAM restorations investigated in this research study along with the general characteristics, are highlighted in Table 1.

### 2.1. Indications/Technological Considerations

All CAD polymeric materials have the indication of long-term provisional restorations, although the term “long term” is a relative one and not clearly defined. Furthermore, the time needed for a provisional restoration is variable, between 6 and 8 weeks up to a month or 1–2 years, depending on the reconstruction of the adjacent maxillofacial structures.

### 2.2. Indentation Experiments

The mechanical properties at nanoscale (micro-elastic modulus (E) and micro-hardness (H)) of all CAD/CAM restorative materials involved in the study were investigated by nanoindentation using a nanoindenter test system (G200 Keysight Technologies, Santa Rosa, CA, USA). The continuous hardness and elastic modulus with indentation depth can be obtained through the continuous stiffness measurement (CSM) technique [17,18]. In the CSM method, the indenter tip approaches the surface with an approach velocity of 10 nm/s. When the indenter touches the surface, it starts to penetrate the surface at a rate of 0.05/s and until it reaches the maximum penetration depth of 500 nm; at this point the load on the indenter is held constant for 10 s, in order to eliminate the time-dependent behavior of the material [19], followed by its slow withdrawal from the sample. When the load on the sample reaches 10% of the maximum load, it is held constant for 100 s. Subsequently, the indenter is completely withdrawn from the material, and the sample is moved for the next test.

The indentation tests were carried out on cube specimens with a size of 10 mm × 10 mm × 5 mm at room temperature, using a triangular pyramid Berkovich diamond indenter (the tip radius ≤20 nm) and performing 15 indentations with 50 µm distance between them (to prevent interactions between indentations) for each sample. The maximum indentation depth was set to 500 nm with the displacement indentation tests of 10 nm/s, strain rate of 0.05/s and thermal drift of 0.05 nm/s. According to previous researchers [19,20] the penetration depth should be enough to minimize the surface effect (more than 200 nm for polymeric materials) and, at the same time it should be small enough to avoid specimen damage and substrate effects. In this context, the selected 500 nm indentation depth will fulfill all these requirements.

### 2.3. Statistical Analysis

Statistical analysis of the data was performed with commercially available GraphPad Prism Software, Version 8.0.1, two-way ANOVA method, taking a *p* value < 0.05 as statistically significant. The data were expressed as mean ± SD, *n* = 3.

## 3. Results

### 3.1. Load-Displacement Curves

**Figure 2:**
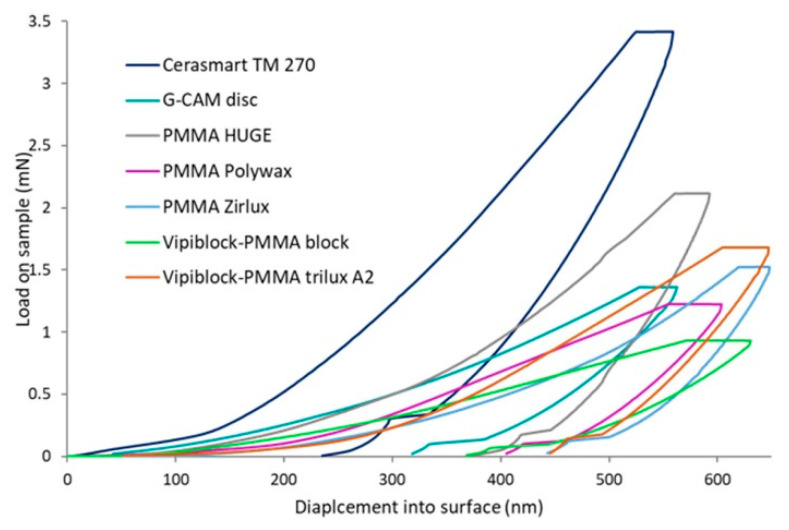
Graphical representation of load-displacement curves of all investigated CAD/CAM specimens subjected to nanoindentation.

The obtained load-displacement curves of all CAD/CAM restorations indented under the strain rate of 0.05/s at an indentation depth of 500 nm at room temperature are shown in Figure 2.

Generally, the force-depth curves are described by a smooth, continuous, non-linear loading region, characteristic of non-linear materials (e.g., viscoplastic, elastoplastic materials) with no pop-in events followed by the unloading region, in which, besides the smooth non-linear profile, the specific well-defined pop-out phenomena along with elastic increment recovery and the plastic deformation of CAD/CAM dental materials, are observed, regardless of the investigated specimen. Important differences are noted both in the load applied to reach the maximum indentation depth of 500 nm and of the inertial effect during the peak hold time after the indenter has reached the targeted loading value. The lowest nanoindentation force is used for CAD/CAM PMMA-organically-modified ceramics dental material (Vipiblock-PMMA block), the maximum penetration depth is reached at a load of ~1 mN, then, the applied force has an increasing trend with the maximum load applied on restorative CAD/CAM material based on hybrid ceramic, Cerasmart 270 (~3.5 mN), followed by the CAD/CAM material based on multilayered PMMA Huge with ~2.3 mN.

### 3.2. Load during Loading Stage

**Figure 3:**
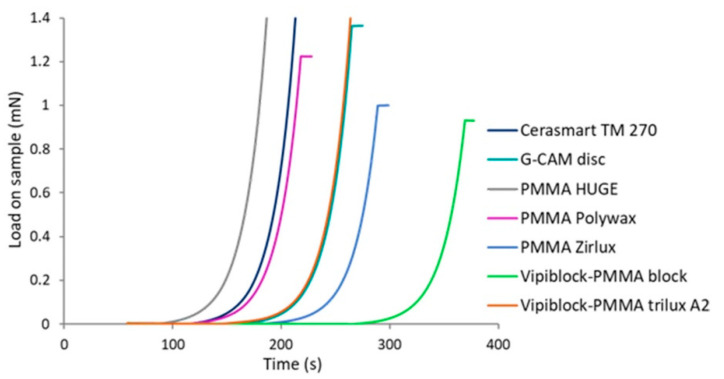
Graphical representation of load as function of time curves at the loading stage of the investigated CAD/CAM restorative specimens under nanoindentation.

The load history of materials during the loading stage at a constant strain rate of 0.05/s applied in the indentation process of CSM method, are investigated and presented in Figure 3.

It is known that the time to reach a certain force can be shortened by increasing the load, and oppositely, a high force is necessary for a high strain rate at the same indentation depth [21]. Considering that the experiments were performed at constant load and the same penetration depth, the variation in the load curves are exclusively originated from the local and respectively intrinsic mechanical properties of materials. Thus, CAD/CAM restorations based on hybrid ceramic (Cerasmart 270) or graphene-based composites (G-CAM disc) will need a high load to reach the penetration depth as compared to CAD/CAM restorative dental materials or composite materials based on PMMA (Vipiblock-PMMA block, PMMA Zirlux).

### 3.3. Continuous Contact Stiffness

**Figure 4:**
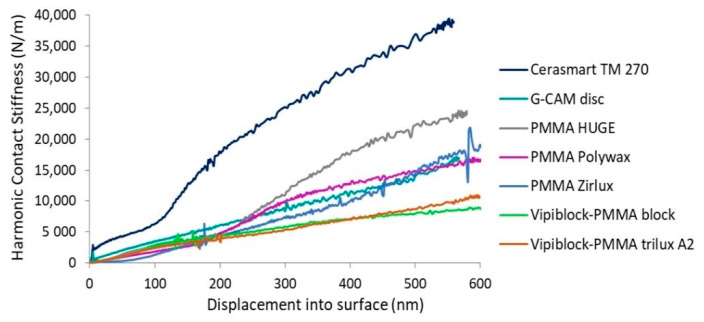
Harmonic contact stiffness as function of displacement of the investigated CAD/CAM restorations under nanoindentation experiments.

It can be observed that the continuous contact stiffness of investigated CAD/CAM restorative materials increases nonlinearly with the increasing depth (Figure 4), which is characteristic of polymer-based composite materials [22].

According to the literature, the contact stiffness is proportional to the root square of the contact area [23]. In other words, the increasing indentation depth during the hold time, where the samples initially present a viscoelastic behavior and then either viscoelastic, viscoplastic one or both, the material continues to strain and the indenter depth still increases, the contact area continues to increase, in correlation with load-displacement curves.

### 3.4. Indentation: Micro-Elastic Modulus and Micro-Hardness

**Figure 5:**
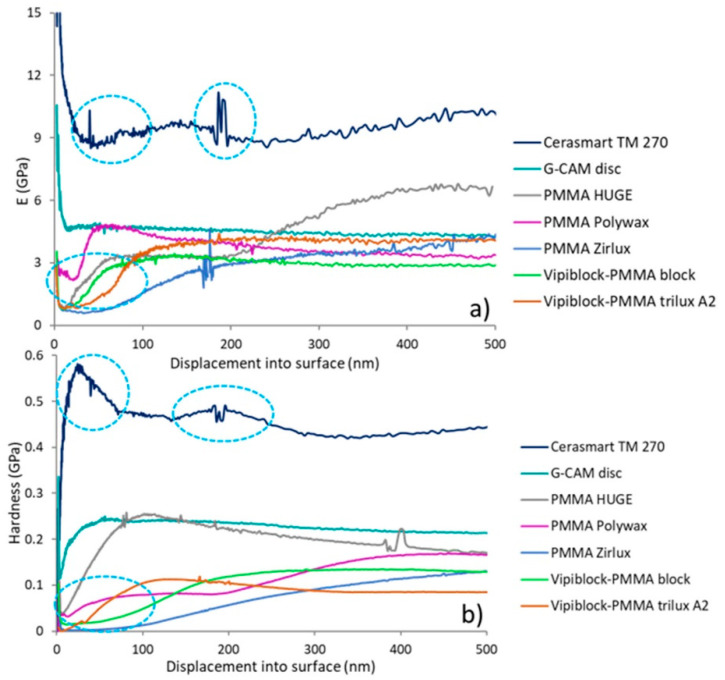
Representative variation of (**a**) micro-elastic modulus (E) and (**b**) micro-hardness (H) as function of indentation depth.

The history of micro-elastic modulus (E) and micro-hardness (H) during the loading stage is presented in Figure 5a,b.

Significant differences in the evolution and shape of curves recorded for both E and H, which are mainly originated from the structure of investigated CAD/CAM restorative dental materials, are noted. The micro-elastic and micro-hardness curves of hybrid ceramic CAD/CAM restoratives present the highest values of micro-mechanical parameters, which are slightly altered during the loading stage, behavior observed in almost all investigated dental materials. Contrastingly, the micro-mechanical features of CAD/CAM restoration based on PMMA-graphene (G-CAM disc) are constant with the indentation depth; regardless of the indentation point, the curves of mechanical features are uniform and straight. Overall, the curves of micro-elastic modulus (E) and micro-hardness (H) decrease with the indentation depth (until the indenter reaches 100 nm), followed by their stabilization beyond the surface’s anomalies.

**Figure 6:**
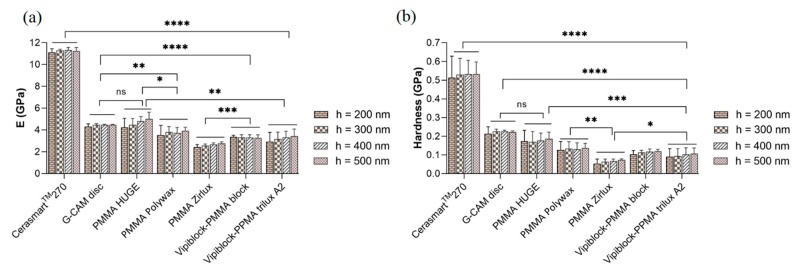
(**a**) Micro-elastic modulus (statistical significance: ns > 0.5; * *p* < 0.05; ** *p* < 0.005; *** *p* < 0.001; **** *p* < 0.0001) and (**b**) micro-scale hardness (statistical significance: ns > 0.5; * *p* < 0.05; ** *p* < 0.01; *** *p* < 0.0005; **** *p* < 0.0001) of all investigated CAD/CAM restorative specimens at different values of indentation depth.

The values of micro-scale elastic modulus and micro-hardness as function of displacement are presented in Figure 6a,b. As already mentioned, the surface contribution (surface anomalies) is reflected in the large standard deviations of the displacement range from 100 to 200 nm of both parameters.

The highest values of micro-elastic modulus and micro-hardness are recorded for hybrid ceramic-based CAD/CAM restoration (Cerasmart 270), ranging between 11.10 and11.23 GPa (*p* < 0.0001) and 0.51 and 0.53 GPa (*p* < 0.0001), followed by the CAD/CAM restorative composite based on polymer-graphene (G-CAM disc) with micro-scale modulus ranging between 4.30 and 4.47 GPa, respectively, micro-hardness between 0.21 and 0.22 GPa. The nanomechanical parameters of multilayer PMMA block CAD/CAM specimen (PMMA HUGE) is in the range of 4.23–5.00 GPa for micro-modulus and between 0.17 and 0.19 GPa for micro-hardness, while the big differences in the obtained values, even at high displacement, may originate from the heterogeneity of material. Overall, the lowest micro-elastic modulus ranging between 2.43 and 2.77 GPa and micro-hardness 0.05–0.07 GPa are recorded for the CAD/CAM restorative dental material based on multilayered PMMA (PMMA Zirlux).

## 4. Discussion

Considering the structural complexity (multicomponent, heterogeneous microstructure) and multifunctionality of dental tissue, the clinical performance of a material with potential application in dental tissue restoration, besides physical and chemical features, is generally driven by its global mechanical (e.g., strength, elastic modulus, hardness) and particularly nano/micro-mechanical characteristics. Among the variety of techniques described and used in the specialized literature, nanoindentation represents an effective method in the assessment of mechanical properties of materials, such as hardness and Young’s modulus on a micro- and nanoscale level, providing accurate and consecutive measurements of the indentation load (P) and penetration depth (h) [24]. Mainly, owing to its advantages, such as high spatial resolution, simplicity and ability to make nano/microscale measurements, this method has become a very powerful approach in accurate investigation of nanomechanical features of dental restorative materials [21,25]. In conventional nanoindentation, an indenter tip is driven into the material surface with a known force and retracted sequentially; during indenting the load and depth of penetration are recorded, which are next used to construct the force–displacement curve [24]. From the load-displacement curve, the mechanical properties, elastic modulus and hardness of the investigated material can then be extracted [24,26]. Thus, the nanoindentation load-displacement curve represents the “mechanical fingerprint” of a material response to contact deformation [27], revealing important information concerning the intrinsic homogeneity of investigated materials as well as the elastic increment recovery and the plastic deformation (Figure 2).

Generally, during the nanoindentation process, the investigated material may be subjected to different stress-induced phase transformation mechanisms, which are reflected in load–displacement curves as three types of phenomena (e.g., pop-ins in the loading process, pop-outs and elbows in the unloading process) (Figure 2) [28]. Pop-ins and pop-outs lead to discontinuities of load–displacement curves, whereas elbows change the slope of the unloading curve sharply. In a load-controlled test, pop-ins are characterized by unexpected bursts of indentation displacement, the first pop-in marking the transition from purely elastic behavior to plastic deformation and the formation of a permanent hardness impression, this event acts as a trigger for the onset of plastic deformation [29,30] and is not observed in the registered load-displacement curves. According to the literature, the pop-out effects, which are observed in the unloading region of all load-displacement curves (Figure 2) have been attributed to many different factors [21,24,25,26,27,28] and mainly depend on the unloading conditions, such as the indentation and unloading rate. Usually, a large indentation load and a low unloading rate will promote the appearance of these effects.

Since nanoindentation investigates the surface mechanical properties at microscale level, besides the global mechanical properties (hardness, elastic modulus) the applied load is strongly dependent on the local microstructural features of the material [31], and is clearly reflected in the discrepancy of applied forces to reach the maximum indentation depth of 500 nm on investigated specimens (Figure 2). It is obvious that CAD/CAM restorations based on hybrid ceramic (Cerasmart 270) or graphene-based composites (G-CAM disc) will need a high load to reach the penetration depth when compared to CAD/CAM restorative dental materials or composite materials based on PMMA (Vipiblock-PMMA block, PMMA Zirlux). During the peak hold time (100 s), after the indenter reached the targeted value of 500 nm (according to the working protocol), the viscoelastic or viscoplastic response of materials along with the viscous behavior of PMMA then cause the indenter depth to increase above the selected value in the load-displacement curves [23]. This inertial effect (also noticed in the continuous stiffness measurements) is visible in all the investigated samples, except the dental materials based on hybrid ceramics (Cerasmart 270) (Figure 2 and Figure 4).

It is well-known that elastic modulus represents an intrinsic property of material and, at a fundamental level, it is a measure of the bond strength between atoms [32]. Unlike elastic modulus, hardness is an engineering property and is defined as the resistance of a material to permanent deformation during application of load. The variation of micro-elastic modulus and micro-hardness is correlated with the heterogeneity of CAD/CAM restorative materials and their relative stabilization with the higher penetration depth may probably be attributed to the decrease of the nanomechanical heterogeneity (Figure 5). At the same time, the preservation of constant micro-mechanical features at different points during the indentation depth, observed in the case of CAD/CAM restoration based on PMMA-graphene (G-CAM disc), represents a particularity of homogeneous materials (graphene layers are in a high dispersed state within the PMMA matrix).

Among the investigated dental specimens, hybrid ceramic-based CAD/CAM restorations (Cerasmart 270) presented the highest micro-mechanical properties, followed by the CAD/CAM PMMA-graphene dental material, and the lowest micro-mechanical features were registered for CAD/CAM multilayered PMMA, while according to the indentation results, CAD/CAM restoration based on PMMA-graphene showed the highest homogeneity. The microstructural homogeneity of materials represents a key-parameter that drives the global mechanical performances of a composite material [33].

Since dental restorations are mainly heterogeneous materials in the micro-/nanoscale scale level, the accuracy and the limitation of the indentation experiment in the mechanical characterization of dental nanocomposites requires further investigation on the mechanism of elastic-plastic deformation under indentation process. Aiming to comprehensively investigate and understand the local and global behaviors of dental materials, Karimzadeh et al. [19] studied the internal behavior and mechanism of deformation of dental composite specimen under the indentation load through combining the nanoindentation experiment with the computational simulation approach (e.g., finite element). Moreover, it should be noted that the present study investigated the mechanical behavior of CAD/CAM restoration specimens under constant loading in standard laboratory conditions. Therefore, comprehensive assessment of the mechanical behavior of dental restorative materials under different loading accompanied by the detailed in vivo biological and technical investigations (e.g., the survival rate, biological response and complications, versatility and ability to satisfy the functional and aesthetic demands) are mandatory to predict their reliability, longevity and clinical performances.

## 5. Conclusions

Nanoindentation has proven to be one of the most precise ways of determining and comparing the micro-mechanical properties of restorative materials. The results of in vitro indentation tests conducted under laboratory standard conditions provided usable and comparable information about the micro-mechanical behavior of investigated commercially available CAD/CAM restorations. The data suggest that hybrid ceramic-based CAD/CAM presented the highest micro-mechanical properties, followed by the CAD/CAM PMMA-graphene, which also presented the highest degree of homogeneity among the investigated dental specimens, while the lowest micro-mechanical features were registered for CAD/CAM multilayered PMMA. Within the limits of this in vitro study, from the micro-mechanical point of view, the hybrid ceramic-based CAD/CAM are more suitable for restorations, however, further clinical investigations need to be performed to investigate their safety and clinical performance as reliable dental materials for provisional or long-term restorations.

## Figures and Tables

**Table 1:** General characteristics of materials employed in the study.

Commercial Name	Composition	Mechanical Characteristics *		Manufacturer/ Country
		Flexural Strength	Elastic Modulus	
Vipiblock—PMMA block	PMMA, EDMA, organically modified ceramics	>100 MPa	>2200 MPa	VIPI Odonto Products Ltd./ Pirassununga, Brazil
Vipiblock—PMMA trilux monocrom A2				
PMMA Zirlux	Multilayered PMMA	>100 MPa	>2200 MPa	Henry Schein, Inc./ Melville, NY, USA
PMMA Polywax	Multilayered PMMA			Bilkim Co. Ltd./ Izmir, Turkey
PMMA HUGE	Multilayer PMMA block	>120 MPa	>2200 MPa	Shandong Huge Dental Material Corporation/Shanghai, China
G-CAM disc	PMMA doped with graphene	>140 MPa	>3200 MPa	Graphenano Dental S.L./ Valencia, Spain
Cerasmart 270	Hybrid ceramic	246 MPa	9600 MPa	GC Corporation/ Tokyo, Japan

* According to manufacturer’s technical sheet.

## Data Availability

The data presented in this study are available on request from the corresponding author.
